# Improving Accuracy of Handoff by Implementing an Electronic Health Record–generated Tool: An Improvement Project in an Academic Neonatal Intensive Care Unit

**DOI:** 10.1097/pq9.0000000000000329

**Published:** 2020-07-10

**Authors:** Jenny K. Koo, Laurel Moyer, Michaela A. Castello, Yassar Arain

**Affiliations:** From the * Division of Neonatology, Department of Pediatrics, University of California, San Diego, La Jolla, Calif.; † Rady Children’s Hospital, San Diego, Calif.; ‡ Department of Pediatrics, University of California, San Diego, La Jolla, Calif.

## Abstract

**Background:**

Written patient handoffs are susceptible to errors or incompleteness. The accuracy is dependent on the person inputting the information. Thus, handoff printouts generated by electronic health records (EHR) with automation reduces the risk of transcription errors and improves consistency in format. This single-center quality improvement project aims to increase the accuracy of handoff printouts with an EHR-generated handoff tool.

**Methods:**

This project used a plan-do-study-act methodology. Participants included registered nurses, neonatal nurse practitioners, neonatal hospitalists, pediatric residents, neonatal fellows, and neonatologists. The goals were to (1) increase accuracy of information to 80%, (2) reduce verbal handoff time by 20%, (3) reduce the frequency of incorrectly listed medications below 20%, and (4) improve user satisfaction by 1 point (on a 5-point Likert scale) over 6 months. Baseline assessment included a survey and a review of handoff reports 4 months before transitioning to the new handoff tool. We created a new handoff tool using EHR autogenerated phrases (Epic SmartPhrases) and autopopulated fields for pertinent Neonatal Intensive Care Unit patient data.

**Results:**

After the unit-wide implementation of the new tool, the accuracy of 16 patient data points increased from 51% to 97%, while the frequency of patients with incorrectly listed medications decreased from 51% to 0%. Handoff time remained unchanged, while a 5-question user satisfaction survey showed an increase on the Likert scale.

**Conclusions:**

We demonstrated that handoff printouts generated by EHR have fewer inaccuracies than manually scripted versions and do not add to the time required to give verbal handoff.

## Abstract 2

Supplemental Digital Content is available in the text.

## INTRODUCTION

Patient handoffs are highly susceptible to errors, including the omission of relevant information, which can lead to medical errors and patient harm.^1^ Verbal handoffs are accompanied by a physical handoff report that is commonly a free-texted document prepared manually by the providers. With manual input of data, especially in teaching hospitals where there are all levels of trainees, there is a significant risk for transcriptional errors.

Hospital systems have implemented multiple strategies to reduce errors and improve patient outcomes, such as applying work-hour limitations to minimize provider burnout and fatigue.^2,3^ However, shorter work hours ultimately led to an increase in the number of handoffs,^2,4,5^ and despite the apparent improvement in resident well-being, they have not demonstrated any significant improvement in patient outcomes.^6–8^ Studies have shown that preventable complications may occur more frequently in patients under the care of a covering physician, suggesting that discontinuity of care and increased handoffs may contribute to adverse outcomes.^9,10^

Other efforts to reduce handoff errors include standardizing verbal handoffs with processes such as the “IPASS” system (illness severity, patient summary, action items, situation awareness and contingency planning, and synthesis by the receiver).^11–13^ The IPASS mnemonic prompts providers to include all critical elements in their handoff. The use of the IPASS system significantly reduced medical errors and adverse events.^11,14^ Standardized procedures, such as the IPASS, can improve handoff effectiveness; however, handoffs are still subject to communication errors when providers rely on verbal or written transcription.

Thus, supplementing standardized verbal procedures with additional handoff tools may further reduce errors. One such tool that could reduce errors is the electronic health record (EHR). There is 15 autopopulation of data from the EHR to a handoff printout. It reduces transcription errors, including mistakes about active medication and doses and essential patient demographics.^15,16^

Our quality improvement initiative aimed to increase completeness and accuracy of handoff sheets through the implementation of an EHR-generated handoff tool with autopopulated fields for pertinent Neonatal Intensive Care Unit (NICU) patient data. The objectives of this initiative were (1) to increase the accuracy of recorded information to 80%, (2) to reduce handoff time by 20%, (3) to reduce the frequency of incorrectly listed medications and dosing on a handoff to 0%, and (4) to improve user satisfaction by 1 point (on a 5-point Likert scale) over 6 months.

## METHODS

The University of California, San Diego, Jacobs Medical Center is a level 3 NICU academic center with residents, fellows, attendings, neonatal nurse practitioners (NNP), and hospitalists, all of whom use the handoff tool built within the hospital system-wide commercial EHR (Epic Systems, Verona, WI). The NICU is a 49-bed unit with an average census of 28 patients during the duration of this project. There are 2 clinical teams: the first includes a neonatologist, a neonatal fellow, and 2–3 pediatric residents, and the second consists of a neonatologist and 1–2 NNPs and/or hospitalists.

**Fig. 1.**
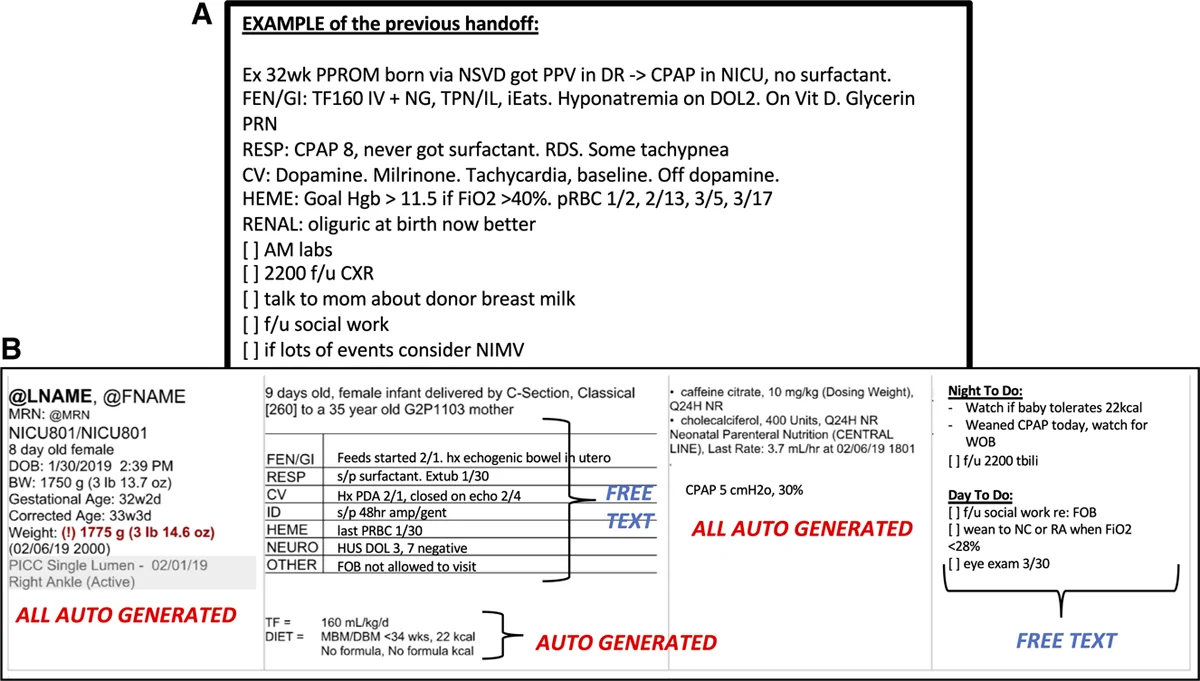
Examples of the handoff tools before and after implementing changes. A, The old, existing handoff tool was a textbox with no structure, prompts, or autogenerated data. B, The new handoff tool features well-demarcated sections (demographics, patient problem list, medications list, respiratory support, daytime to-do list, and nighttime contingency plans). Note that much of the critical data is autopopulated by the EHR.

**Fig. 2.**
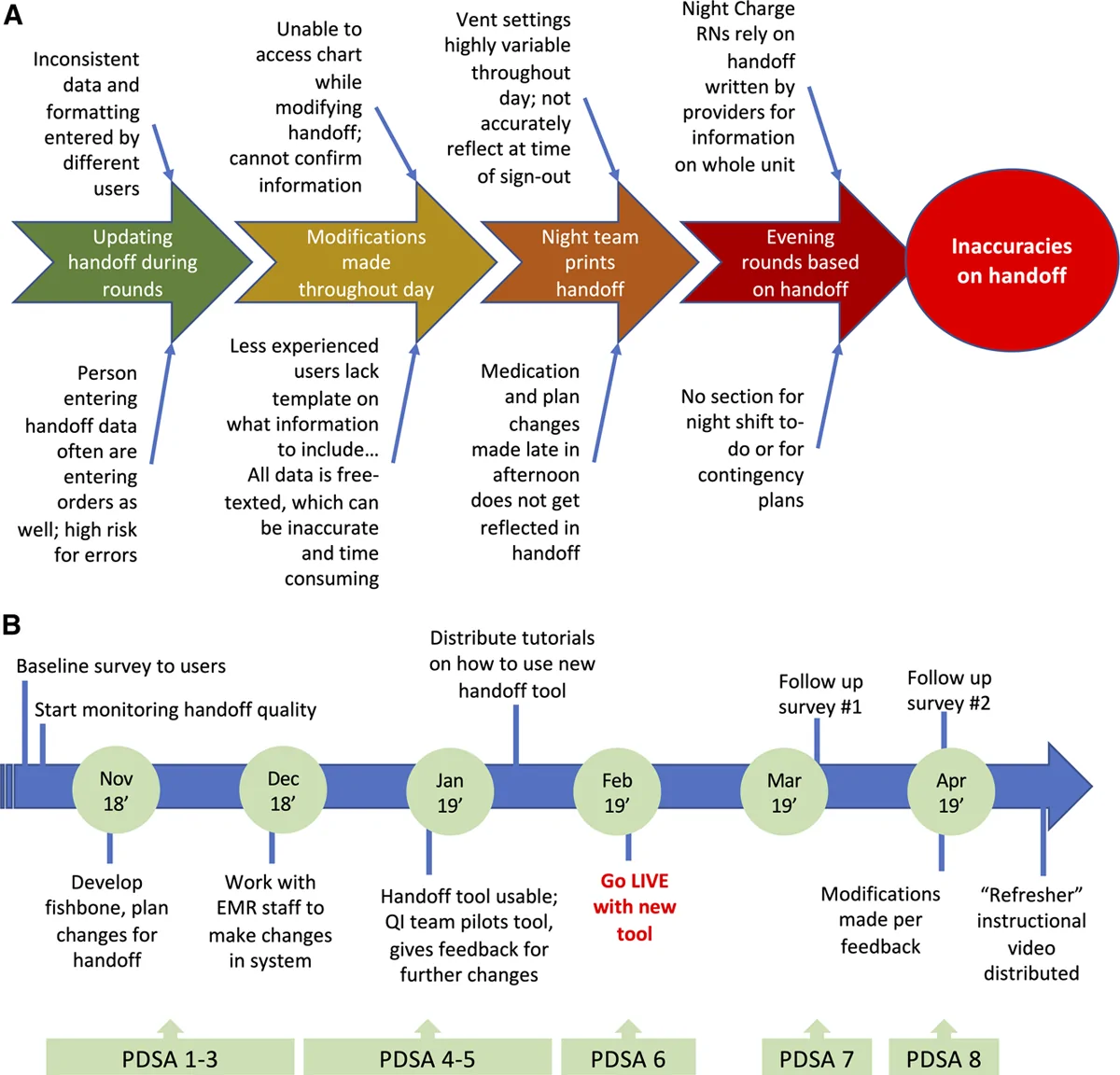
Quality improvement project work flow. A, This modified Ishikawa diagram depicts the contributing factors that make the prior handoff tool prone to error and incompleteness. B, Timeline of the quality-improvement project.

A cross-covering fellow and a pediatric resident staff the entire unit at night. The verbal handoff from the day team to the night team is supplemented by a printed handoff tool that serves as the patient census list as well as a quick reference for patient information if a computer is not immediately available for access to the EHR. Additionally, charge nurses use this handoff for information necessary for nursing assignments and determining nurse-to-patient ratios. Charge nurses reference but do not edit the handoff. At this facility, the old handoff tool was a textbox with no format, structure, or prompts to the user for what data should be manually included (Fig. 1A). Depending on the user, the handoff structure could vary considerably and be often prone to missing or erroneous data. The Ishikawa diagram (Fig. 2A) summarizes the contributing factors in a day-to-day NICU workflow that contributes to inaccuracies or missing data in handoffs.

NICU fellows, attendings, residents, NNPs, and charge nurses provided input to create a new handoff tool using EHR autogenerated fields for pertinent NICU patients. Each plan-do-study-act (PDSA) cycle involved drafting the content, layout, and EHR autogenerated elements. We presented each draft to various stakeholders by creating handoff printouts of 2–3 mock patients using the draft handoff template. Feedback from stakeholders was studied and accounted for with each revision of the handoff tool. The primary project team members created the first draft. We presented the first draft to charge nurses for feedback in PDSA cycle 1, and we agreed to incorporate autogenerated diet order into our revisions. In PDSA cycle 2, we presented the draft to NNPs and hospitalists, and we agreed upon a portrait orientation of the printout to allow up to 4 patients to be printed on a single sheet. In PDSA cycle 3, the revised draft was presented to the Epic EHR specialist, who gave feedback on what was and was not feasible through EHR programming. Data that could not be automatically generated by the EHR remained as manually inputted text fields. The resulting final version is depicted in Figure 1B.

The elements of patient demographics required on the handoff include patient name, room number, medical record number, date of birth, birth gestational age, corrected gestational age, birth weight, and current weight. Critical elements of patient medical information include medications (including doses and frequency), ventilator or respiratory support level and settings, total fluids, diet order, central line access, overnight contingency, overnight laboratories and images, and daytime to-do list. These items were mostly consistent with elements included in the NICU handoff tools at other institutions.^15^

The project spanned from October 2018 to May 2019 (Fig. 2B). The monitoring of handoff quality began in October 2018. A baseline survey was distributed at the start of the project to assess user satisfaction. Patient handoffs were audited 4–5 times per month, with each audit reflecting a single “sample.” The scorable elements of patient demographics, medical data, and medication information are as listed above. By late January 2019, we distributed video and slide show tutorials as PDSA cycle 4, and we welcomed any additional feedback or questions from users before launching the new tool. A pilot run (PDSA cycle 5) tested the new handoff tool’s usability on 5 real patients, with mock verbal handoffs between team leads. The “go-live” date for the new handoff tool was in February 2019 (PDSA cycle 6). Two additional surveys spaced 1 month apart were distributed as PDSA cycles 7 and 8 to elicit any feedback and revisions necessary to streamline the new handoff tool. Some of the proposed revisions included improvements made to the autogenerated ventilator settings section and changes made to the layout of the systems-based problem list.

The primary measurable outcome was improved handoff accuracy and completeness to 80% accuracy in the composite of demographic and medical data. The goal of 80% overall accuracy is practical as it allows for any errors resulting from areas in the handoff that still require manual input. On the other hand, the frequency of incorrectly listed medications should be reduced entirely to 0% since the medication list is entirely autogenerated by the EHR.

As a balancing measure, the time required to complete verbal handoff was recorded (in minutes) to reduce this time by 20%. An online survey measured user satisfaction of the handoff tool before publishing the new handoff tool and at monthly intervals after implementation.

Satisfaction scores were measured on a 5-point Likert scale, and a free text field permits entry of additional feedback and suggestions. Our goal to increase the satisfaction score by 1 point reflects a 20% improvement. It was a realistic target, given the high probability that workflow changes are often met with some resistance.

Process measures involve ensuring 100% compliance with the new handoff tool by switching the system over in Epic on a designated “go-live” date. This change was monitored through Epic chart audits before and after the new handoff tool “go-live” date.

Outcome measures are analyzed using statistical process control displayed on control U charts. The analysis of these measures adhered to rule-based conventions for special cause variation, as defined by Provost and Murray.^17^ For patient demographics, every patient has 8 scorable elements, so the denominator for each audit is the product of 8 and the census. For patient medical information, the denominator is the total number of scorable elements pertinent to the patients. For medication details, the denominator is the total number of patients at the time of handoff auditing, and the number of events was the number of patients with at least 1 missing or incorrect piece of information (medication name, dose, frequency, and route). (See Figure 2, Supplemental Digital Content, which displays sample handoff audit scorecard for a mock unit census of 3 patients, http://links.lww.com/PQ9/A202.)

Upon discussion with the University of San Diego Human Research Protections Program (HRPP) staff, this quality-improvement project did not qualify as human subject research, and therefore, it was exempt from Institutional Review Board (IRB) review. Patient rights to privacy remain protected, and the parties involved in the project have access to patient information through direct medical care. This project poses no risks to patient safety.

## RESULTS

**Fig. 3.**
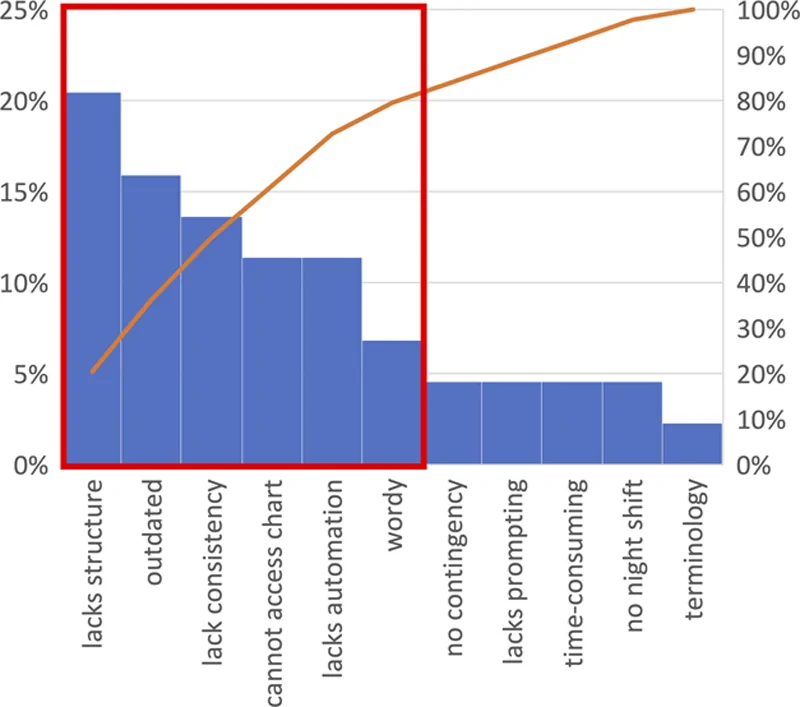
Free text responses in the baseline survey from 35 respondents are analyzed for frequent key terms. The Pareto chart depicts the most common responses. The critical contributors to handoff dissatisfaction at baseline include the old handoff tool’s lack of structure, outdated information, lack of consistency, inability to access the patient chart while editing the handoff, the lack of automation, and the wordiness of free-texted manually typed handoffs.

Thirty-five providers completed a baseline survey, including NICU fellows (9%), residents (37%), attendings (3%), NNPs (6%), and charge nurses (45%). Free-text responses were content analyzed for key terms, and Figure 3 depicts the frequency of key terms in a Pareto chart. The critical contributors to handoff dissatisfaction at baseline include the old handoff tool’s lack of structure, outdated information, lack of consistency, inability to access the patient chart while editing the handoff, the lack of automation, and the wordiness of free-texted manually typed handoffs.

We achieved a 100% compliance rate on the go-live date since the new handoff tool entirely replaced the old handoff tool on the EHR, so providers were mandated to use the new handoff tool.

**Fig. 4.**
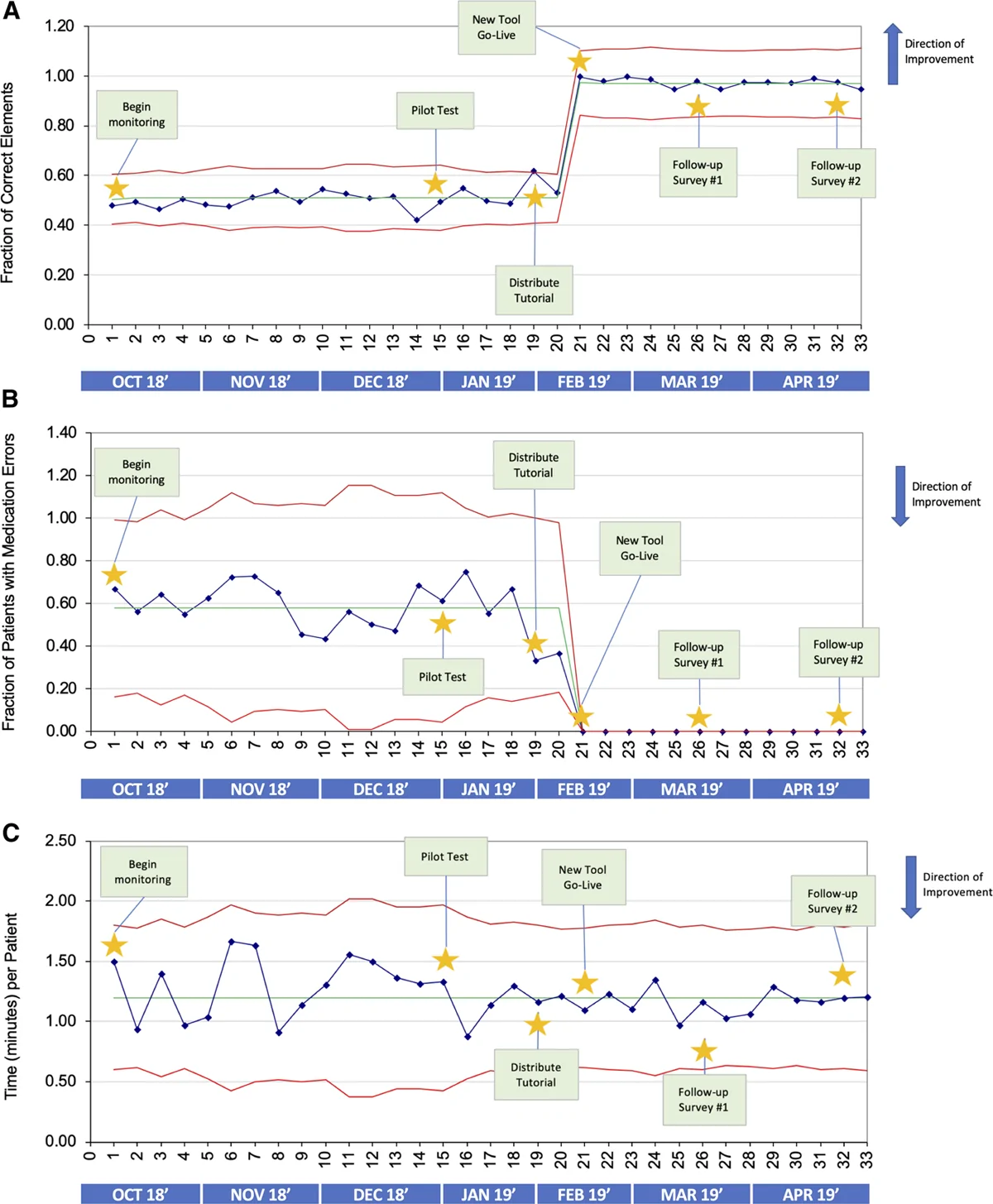
Statistical process control chart (U charts) demonstrates improvement in patient data accuracy in multiple categories after implementation of the new handoff tool. A, Patient composite data (demographics + medical information) accuracy improved from 51% to 97%. B, Statistical process control chart (U charts) demonstrating a reduction in the frequency of patients with incorrect or incomplete medication lists from an average of 51% to 0% after implementation of the new handoff tool. C, The control chart of time (in min) spent per patient during evening verbal handoff demonstrates no signals.

Figure 4A depicts the control U charts for accuracy of the composite data points (including patient demographics and medical data) over 7 months. After implementing the new handoff tool, the composite accuracy of all patient data points demonstrated a significant shift in the direction of improvement. Demographics and medical information both individually show substantial improvement in accuracy with the addition of the new handoff tool, with a shift seen in our centerline showing significant improvement (see Figure 1, Supplemental Digital Content, which displays (A) patient demographics accuracy improved from an average of 37% to 100%, and (B) patient medical data accuracy improved from an average of 66% to 94% after implementing the new handoff tool, http://links.lww.com/PQ9/A202). Sample No. 19 was outside of the upper control limit preintervention, which indicates special cause variation. Upon further investigation, that sample was likely an outlier that resulted from improved data accuracy after a diligent fellow carefully reviewed and modified every handoff to ensure completeness. This signal indicates that an isolated factor independent of the changes implemented by this quality-improvement project temporarily affected the results.

Figure 4B represents the frequency of patients with incomplete or incorrect medication lists on the handoff. A notable shift occurs when implementing the new handoff tool to 0% frequency of medication information error as this field on the new handoff tool is entirely automated. There was no improvement in the time required for a verbal handoff during this quality-improvement process (Fig. 4C).

**Fig. 5.**
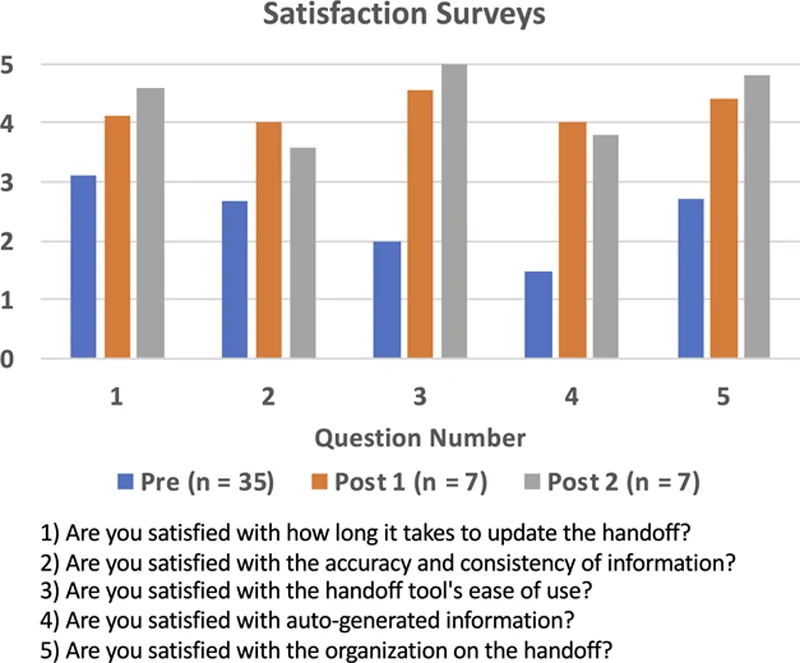
This user-satisfaction survey assesses the baseline satisfaction with the old handoff tool and twice after the new tool was launched. The questions are ranked on a 5-point Likert scale.

User satisfaction appeared to increase after implementation of the new handoff tool (Fig. 5) with 7 respondents (3 residents, 2 fellows, and 2 NNPs) in each of the 2 postimplementation surveys. The most common feedback from the qualitative responses was that the users appreciated the clean layout, the autopopulated fields, and the ability to access the patient chart. At the same time, the handoff editing tool was open.

The main challenge we faced with implementing a new computer-based tool was training the involved parties to use the tool correctly and effectively. Those with less experience with computers, or those who are less adept with the EHR, were prone to struggle with finding the tool or printing out the handoff. We addressed these issues by sending periodic emails with tip-sheets and/or instructional videos. Despite initial resistance from some providers, the results indicate that users were satisfied with the tool and found it easy to use. Furthermore, we implemented the new tool when the unit census was low, so providers had greater ease with adapting to the new tool.

## DISCUSSION

This single-center quality-improvement project demonstrated the efficacy of an EHR-generated handoff report to reduce printed handoff errors and incompleteness. Patient demographics, medical information, and medication lists all significantly improved in accuracy after the implementation of the new handoff tool. Additionally, the new tool neither decreased nor increased handoff time, suggesting that it did not add extra burden or interfere with the unit’s workflow. The quality of verbal handoff is not included in the analysis. Although the time required for verbal handoff did not decrease, it was deemed by the baseline survey that time spent on handoff was not among the critical components (Fig. 3). We were able to obtain buy-in from multiple frontline users, including nurses, NNPs, hospitalists, residents, and neonatology attendings and fellows, to achieve 100% compliance.

This quality-improvement process serves as an example of the successful implementation of a new computer-based tool that harnesses the power of technology to improve patient care and safety. Key elements that contributed to this project’s success were (1) buy-in from all involved parties, (2) preparation and distribution of tutorials before the implementation of the new tool, and (3) sustainability of this EHR tool without the need for continued oversight by the quality-improvement team.

Patient safety depends heavily upon handoff’s accuracy, especially when patients are under the care of a cross-cover provider or during the night shift when there is a lower provider-to-patient ratio in the unit.^9^ EHR-generated handoff printouts remove manual transcription errors from the equation and allow for completeness of information without adding extra stress to the providers.^16^ This single-center quality-improvement project, which incorporated an EHR-generated handoff, was successfully implemented in 6 months and achieved the primary aim of improving handoff accuracy. Although no patient outcome differences were measured before and after the implementation of the new tool, there is already a known strong correlation between the accuracy of handoff data and patient outcomes.^15^

There are limitations to this project and its applicability to other centers. While this tool may be directly applicable to any institution using this same EHR platform, this exact tool may not be feasible on a different platform. Moreover, a crucial component of the IPASS handoff structure is “illness severity.” However, this element is still something that has to be manually inputted and/or verbally passed from provider to provider.^11^ We are limited by the lack of a validated neonatal version of the pediatric early warning sign or a neonatal acuity scoring system. “Illness severity” will remain a subjective assessment and cannot be automatically populated into the EHR-generated handoff.

## CONCLUSIONS

In conclusion, designing and implementing an EHR-generated handoff tool with autopopulated patient data fields can reduce handoff error and allow for improved structure and standardization of handoffs. These results and the fact that this project achieved 100% compliance indicate that this initiative is implementable with minimal effort in other units that use an EHR. This study should encourage units that use EHRs to move toward automation in handoff data as these improvements can have a direct impact on patient safety.

## Supplementary Material


