# Supplementary figures and images for: Improving Accuracy of Handoff by Implementing an Electronic Health Record–generated Tool: An Improvement Project in an Academic Neonatal Intensive Care Unit
Source: Pediatr Qual Saf. 2020 Jul 10;5(4):e329. doi: 10.1097/pq9.0000000000000329 (PMC7360222; doi:10.1097/pq9.0000000000000329)

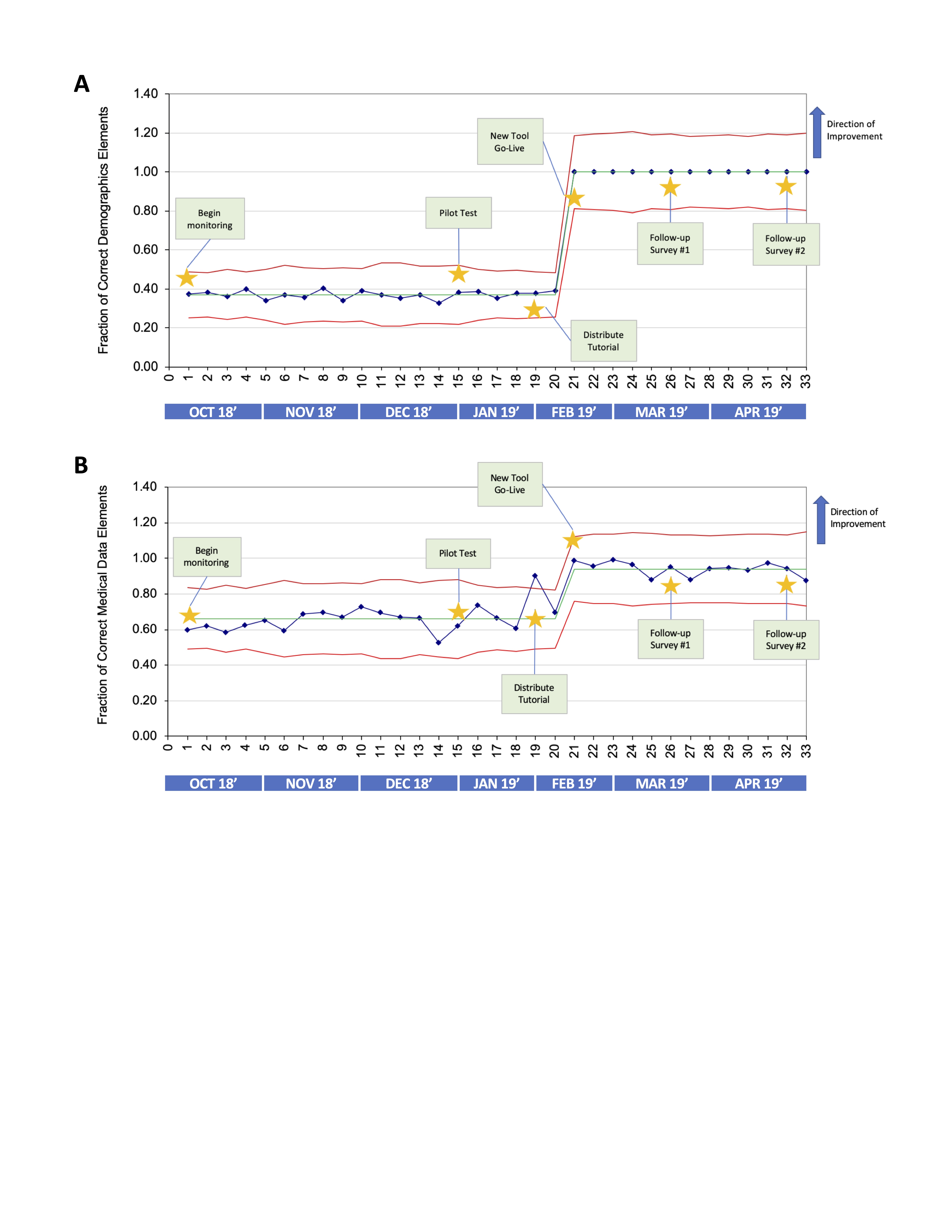

Supplement: Supplementary file 1 [file pqs-5-e329-s001.jpg]

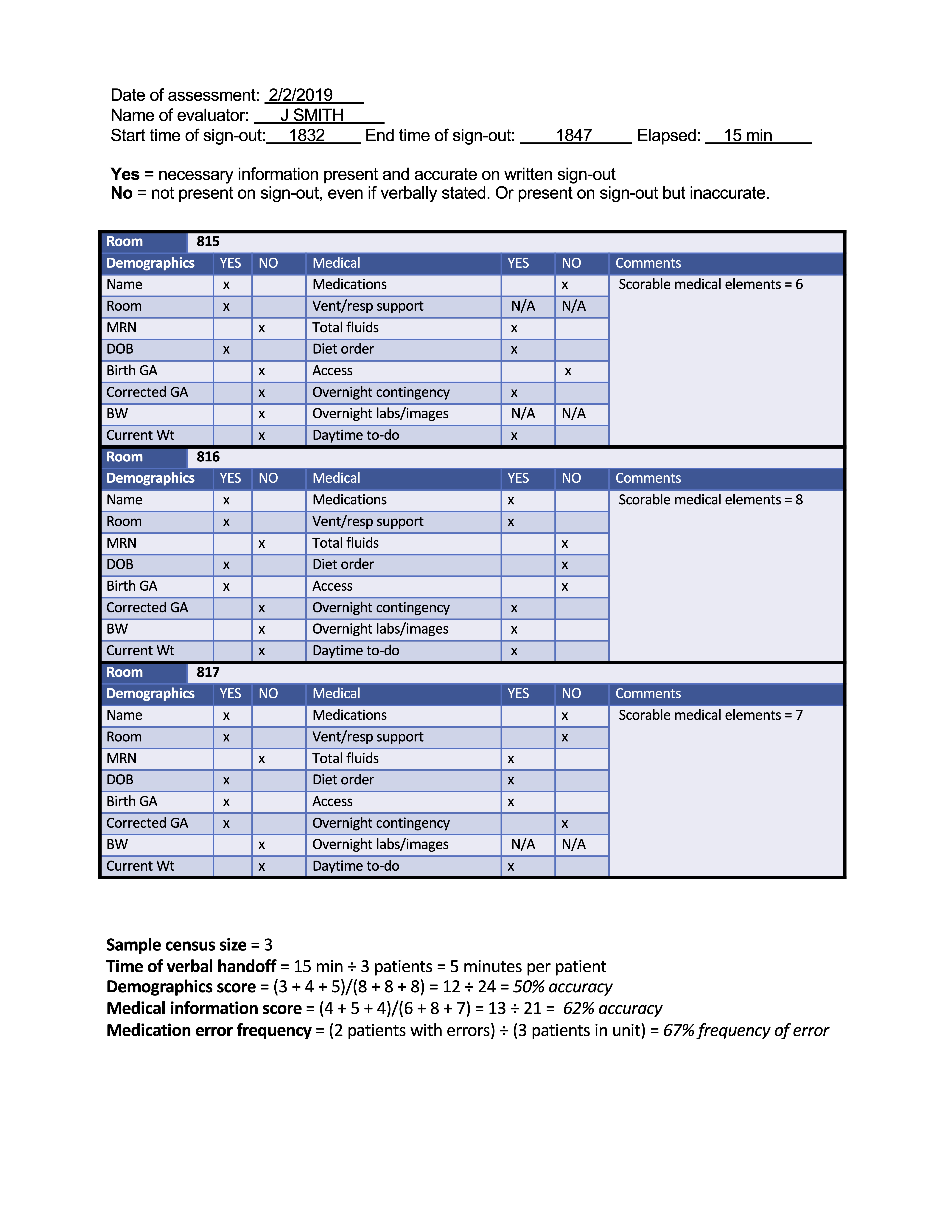

Supplement: Supplementary file 2 [file pqs-5-e329-s002.jpg]
